# SARIMA Model Forecasting Performance of the COVID-19 Daily Statistics in Thailand during the Omicron Variant Epidemic

**DOI:** 10.3390/healthcare10071310

**Published:** 2022-07-14

**Authors:** Khanita Duangchaemkarn, Waraporn Boonchieng, Phongtape Wiwatanadate, Varin Chouvatut

**Affiliations:** 1 Ph.D. Program in Biomedical Engineering, Biomedical Engineering Institute, Chiang Mai University, Chiang Mai 50200, Thailand; kkhanita.du@up.ac.th; 2 Graduate School, Chiang Mai University, Chiang Mai 50200, Thailand; 3 Department of Pharmacy Practice, School of Pharmaceutical Sciences, University of Phayao, Phayao 56000, Thailand; 4 Center of Excellence in Community Health Informatics, Faculty of Science, Chiang Mai University, Chiang Mai 50200, Thailand; varinchouv@gmail.com; 5 Faculty of Public Health, Chiang Mai University, Chiang Mai 50200, Thailand; 6 Department of Community Medicine, Faculty of Medicine, Chiang Mai University, Chiang Mai 50200, Thailand; pwiwatanadate@gmail.com; 7 Department of Computer Science, Faculty of Science, Chiang Mai University, Chiang Mai 50200, Thailand

**Keywords:** time-series forecasting, seasonal ARIMA, SARIMA, COVID-19, coronavirus, predictive modeling

## Abstract

This study aims to identify and evaluate a robust and replicable public health predictive model that can be applied to the COVID-19 time-series dataset, and to compare the model performance after performing the 7-day, 14-day, and 28-day forecast interval. The seasonal autoregressive integrated moving average (SARIMA) model was developed and validated using a Thailand COVID-19 open dataset from 1 December 2021 to 30 April 2022, during the Omicron variant outbreak. The SARIMA model with a non-statistically significant p-value of the Ljung–Box test, the lowest AIC, and the lowest RMSE was selected from the top five candidates for model validation. The selected models were validated using the 7-day, 14-day, and 28-day forward-chaining cross validation method. The model performance matrix for each forecast interval was evaluated and compared. The case fatality rate and mortality rate of the COVID-19 Omicron variant were estimated from the best performance model. The study points out the importance of different time interval forecasting that affects the model performance.

## 1. Introduction

### Background

The outbreak of COVID-19 that began in late 2019 has had a worldwide impact on all facets of society. Although the pandemic has had myriad effects, one of the most easily measured and impactful metrics is the number of confirmed cases of infection. The situation severity is higher when the number of confirmed cases exceeds local healthcare capacity [1]. Protection measures such as wearing a mask, washing hands, and social distancing are still in practice, but the rate of infection does not seem to decrease [2]. In Thailand, the first COVID-19 case was reported in Bangkok on 31 January 2020. With prompt and effective disease control management, the domestic outbreak was controlled by the government and involved high engagement and cooperation from Thai people throughout the private and public sectors [2]. The first cluster began at a boxing stadium and some entertainment venues in Bangkok on 6 March 2020. Seven days after detection of the initial cluster, daily COVID-19 confirmed cases climbed into the 100s. Daily case counts dropped to below 10 per day and the first wave was over by the end of April 2020. The government responded to the first outbreak by declaring a strict policy of disease outbreak control and increasing the level of disease control at international points of entry such as airports, seaports, and land borders. The Centre for COVID-19 Situation Administration was formed by the Ministry of Health to help relieve and manage the critical situation that was caused by the COVID-19 outbreak. A nationwide lockdown was announced after the first cluster was identified [3]. The implementation of mHealth technology and related mobile applications have been used as a tool to help prevent and investigate the outbreak [2]. With multiple modes of outbreak control and a high level of participation from Thai people, daily new confirmed cases in Thailand remained low until the end of November 2020. After April 2021, the Delta variant rapidly spread and dominated the Alpha variant, entering the country into an emergency situation. The healthcare systems were overwhelmed, and the mortality rate rose. The Delta variant confirmed cases gradually declined 4 months after reaching their peak [3,4].

**Figure 1:**
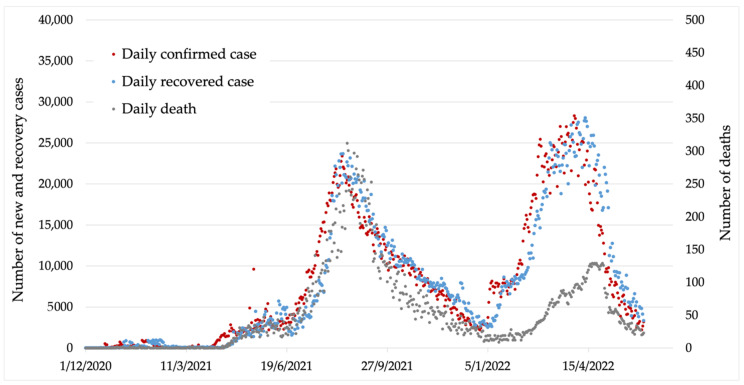
Graphical presentation of COVID-19 cases statistics in Thailand showing the data points used in the study (from 1 December 2021 to 28 May 2022).

Thailand reported the first Omicron cluster on 24 December 2021. The Omicron variant broke Thailand’s record of the highest number of confirmed cases, which continued to rise. However, the case fatality rate during the Omicron outbreak seemed to be lower than in the previous outbreak [5]. Figure 1 shows the dynamics of the daily COVID-19 outbreak from the beginning to 28 May 2022.

The Omicron variant is a variant of concern, according to the WHO announcement on 26 November 2021. There is an increased risk of reinfection with Omicron when compared to the other variants of concern. While the symptom severity of those that are infected with Omicron is unclear, the rapid increase in the Omicron variant confirmed cases is likely to significantly affect public health preparedness in the long run [6].

The number of confirmed cases is consistent with the number of hospitalized and critically ill patients that need respiratory ventilator machines. Dramatic increases in the number of COVID-19 cases without early information or notification can cause shortages in medical facilities and personnel [7]. The most efficient ways to manage the medical workforce at the frontline of the unforeseeable emerging disease outbreak and the needs of medical staff should be considered. Brown-Johnson et al. reported five independent needs of medical staff during the COVID-19 outbreak, including: (1) effective infrastructure and tools for infection control measures amongst healthcare workers on the frontline; (2) effective information sharing about the current situation, policy, and protocols; (3) sufficiently resourced staffing through detailing and cross-training; (4) the nationwide conciliation of human resource management; (5) a mental health support service [8]. To better manage the clinical settings during the COVID-19 outbreak, daily confirmed case forecasting is crucial information for current situation assessment and planning. This information is helpful for policymakers to increase preparedness, such as medical facilities, medication, and notifying medical staff at the frontline, while the most important thing is to avoid a medical supply shortage [9].

## 2. Prior Work

Epidemic forecasting has always been challenging, especially for real-time epidemic forecasting using real-time digital disease surveillance data [10,11,12]. Many studies focus on time-series forecasting the number of cases and the severity of past and current emerging and re-emerging viral infections [13,14,15,16]. 

Lee et al. reported the forecasting of COVID-19 confirmed cases using the autoregressive integrated moving average (ARIMA) method and compared it with the actual confirmed cases [15]. The dataset was divided into five groups based on the peak of the first wave, the period when the increasing confirmed cases rose from their minimum, and the period when there was a variability of confirmed cases that was slightly high before the peak of the second wave, during the peak of the second wave, and the period when there was a variability of confirmed cases that was high before the peak of the third wave. The study analyzed group and case-by-case prediction using the ARIMA model, which accurately determines the increasing and decreasing trends of cumulative numbers of COVID-19 cases in Korea. The ARIMA model performed best at predicting the number of confirmed cases at the time interval of 5 days [15].

Verma et al. reported a model based on ARIMA and fuzzy time series methodology for the forecasting of COVID-19 cases, deaths, and recoveries in India [17]. The model can forecast the value for up to 7 days. The investigators also reported three constraints that caused fluctuation within the forecasted values, including: (1) the external factors that directly affected the dataset such as lockdown and testing capacity; (2) transportation between borders resulting in the numbers of imported cases; (3) asymptomatic individuals not being reported to the healthcare system. 

Gebretensae YA and Asmelash D analyzed trends and forecasted the spread of COVID-19 for developing an appropriate statistical forecast model using the COVID-19 dataset that was reported from the Ethiopian Public Health Institute (EPHI) from 13 March to 31 August 2020 [18]. The auto-regressive integrated moving average (ARIMA) model was used to forecast the confirmed cases, recovered cases, and deaths in Ethiopia. The limitation of the study was that no risk factor was evaluated and analyzed, including the demographic details of patients, nor their social networks and where they had traveled, due to the lack of individual-level data. Some covariates were considered to improve the model performances, such as state or district level daily case data, or the daily confirmed cases in a neighboring country. The results show that using smoothed data and independent covariates significantly improved the model accuracy [19,20]. 

Lucic et al. reported the implementation of the k-mean clustering technique to classify the prediction area based on socioeconomic characteristics within the United States. The ARIMA and seasonal trend random walk forecast were applied to define the pattern of COVID-19 infection rate and mortality. This study shows that the sub-national socioeconomic characteristics play an important role in predicting the infection rate and mortality rate, especially in a country with a higher socioeconomic gap presence [21].

The auto-regressive integrated moving average (ARIMA) model, also known as Box–Jenkins methodology, is the model that uses an iterative approach to identify the most appropriate model with or without a fixed set of predictors from a general model class. The ARIMA model has been proven to effectively forecast the number of new confirmed COVID-19 cases in many countries [20,22,23]. The dataset that can be effectively forecasted using the ARIMA model needs to be stationary, without trends or seasonality. In most cases, the time series of COVID-19 confirmed cases can be made stationary using the differencing method. However, when the trend or seasonality is strongly observed within the time-series dataset, only the differencing method is unable to make the time-series data stationary. In this case, the seasonal ARIMA (SARIMA) model is used because of its ability to model a wide range of seasonal data. However, the challenge of this forecasting method is the hyperparameter estimation for ARIMA and SARIMA models.

This study aims to identify the best seasonal autoregressive integrated moving average (SARIMA) model, and compare the model performance at 7-day, 14-day, and 28-day forecasting of the predictive model for COVID-19 daily statistics during the Omicron variant epidemic in Thailand.

## 3. Materials and Methods

### 3.1. Data Processing

In this study, a total of 151 data points ranging from 1 December 2021 to 30 April 2022 were captured during the Omicron variant epidemic in Thailand. The first reported case of Omicron in Thailand was announced on 24 December 2021. The 3 categories of dataset were retrieved from Open Government Data of Thailand and Thailand National Center of Disease Control, which were open for public analysis, including: (i) the daily number of confirmed COVID-19 cases (domestic cases only); (ii) COVID-19-related deaths; (iii) patients recovered from COVID-19 infection [3,4]. 

**Figure 2:**
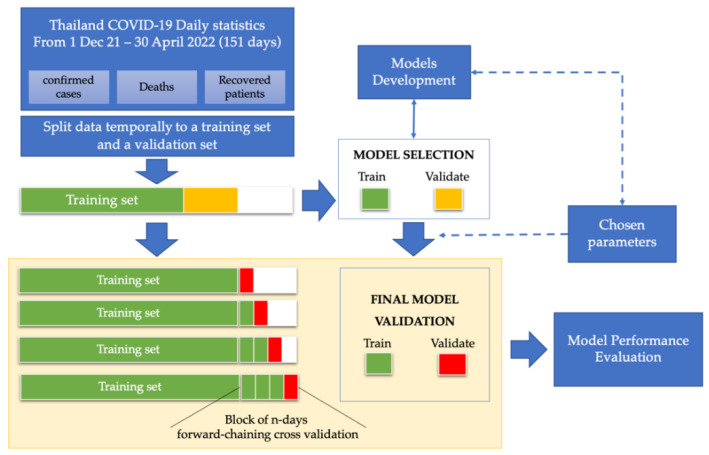
Model validation using day forward-chaining cross validation method.

A total of 151 data points were split temporally into 2 sets: (i) the training set (123 days from 1 December 2021 to 2 April 2022); (ii) the validation set (28 days from 3 April to 30 April 2022). The validation set was temporally split into 7-day, 14-day, and 28-day forecast intervals to compare with the forecasted value. The flow of the data analysis and model validation method is represented in Figure 2.

To visualize the accuracy of the forecasted values, the case fatality rate and mortality rate were calculated and plotted against the observed values.

The case fatality rate (CFR) estimates from the proportion of deaths among identified confirmed COVID-19 cases using the equation [24]:

(1) $$\mathrm{CFR}\;\left(\%\right)=\frac{\mathrm{Number}\;\mathrm{of}\;\mathrm{confirmed}\;\mathrm{deaths}\;\mathrm{related}\;\mathrm{to}\;\mathrm{COVID}\text{-}19}{\mathrm{Number}\;\mathrm{of}\;\mathrm{confirmed}\;\mathrm{cases}}\;\times \;100$$

The mortality rate (MR) estimates using the equation:

(2) $$\mathrm{MR}\;\left(\%\right)=\frac{\mathrm{Number}\;\mathrm{of}\;\mathrm{confirmed}\;\mathrm{deaths}\;\mathrm{related}\;\mathrm{to}\;\mathrm{COVID}\text{-}19}{\mathrm{Thailand}\;\mathrm{total}\;\mathrm{population}}\;\times \;100$$

### 3.2. Model Development and Identification

The ARIMA model was developed using the Python statsmodels package [25] with Spyder 5.0.3, the Scientific Python Development Environment [26]. 

Prior to developing the time-series model, the hyperparameters of ARIMA(p, d, q) and SARIMA(p, d, q),(P, D, Q) need to be estimated and identified. The dataset needs to be validated for the stationary characteristic to verify if it can be performed in the forecasting process using the ARIMA model; otherwise, the SARIMA model is selected. The unit root test or augmented Dickey–Fuller (ADF) test is used in this study to determine if the dataset is stationary, which gives a constant mean and variance over time. Otherwise, the dataset needs to be preprocessed with the differencing method to make the series stationary [27]. 

The degree of difference to make the time-series data stationary is used to identify the ‘d’ hyperparameter of ARIMA(p, d, q). The autocorrelation function (ACF) and partial autocorrelation function (PACF) were applied to the dataset. The hyperparameter AR (p) and MA (q) of the ARIMA(p, d, q) can be estimated using the PACF and ACF diagnostic plots, respectively. The optimal ‘p’ can be obtained from the PACF plot, which describes the correlation of its lagged series. The cut-off range to define the most minimum hyperparameter ‘p’ and ‘q’ in this study were set to a 95% confidence interval. For the final non-stationary time-series data of *X_t_*, the ARIMA (p, d, q) model can be described as follows:

(3) $$\phi (B){\left(1-B\right)}^{d}X_{t}=\psi \left(B\right)\varepsilon_{t}$$

where *d* is the degree of differencing the parameter $X_{t}\;$needs to make the data stationary; *B* is a backshift operator, then $\phi \left(B\right):1-\phi_{1}\left(B\right)-\phi_{2}{\left(B\right)}^{2}-\ldots -\phi_{p}{\left(B\right)}^{p}$ and $\psi \left(B\right):1+\psi_{1}\left(B\right)+\psi_{2}{\left(B\right)}^{2}+\ldots +\psi_{p}{\left(B\right)}^{p}$ are the polynomial expression in *B*; and $\varepsilon_{t}$ denotes the white noise, where, $\varepsilon_{t}\;\sim \;N\left(0,1\right)$. 

In the final step, the dependence between quantities can be described as: $y_{t-n}\;,\ldots ,y_{t-2}$, $y_{t-1}$, $y_{t}$, $y_{t+1}$, $y_{t+2}$, …, $y_{t+n}$. However, if the time-series data contain trends or seasonality, the dependent season can be forecasted as: $y_{t-ns}\;,\ldots ,y_{t-2s}$, $y_{t-1s}$, $y_{ts}$, $y_{t+1s}$, $y_{t+2s}$, …, $y_{t+ns}$, where *s* is a seasonal period. The SARIMA(p, d, q),(P, D, Q) can be formulated as follows:

(4) $$\Phi_{P}\left(B^{s}\right)\phi_{p}\left(B\right){\left(-B\right)}^{d}{\left(B^{s}\right)}^{D}y_{t}=\theta_{q}\left(B\right)\Theta_{Q}\left(B^{s}\right)\varepsilon_{t}$$

where $\phi_{p}\left(B\right)$ is an autoregressive operator, $\theta_{q}\left(B\right)$ is the operator of moving averages, $\Phi_{P}\left(B^{s}\right)$ is a seasonal autoregressive operator, $\Theta_{Q}\left(B^{s}\right)$ is a seasonal operator of moving averages, and $\varepsilon_{t}$ denotes the white noise, where, $\varepsilon_{t}\;\sim \;N\left(0,1\right)$.

The coefficients that were estimated from the selected ARIMA/SARIMA model were based on the best-fitted model between the forecasted data and the testing data. After defining the possible hyperparameter (p, d, q) and (P, D, Q), the range of the hyperparameter was set at two steps forward and backward. The hyperparameter grid search was performed and ranked by the Akaike information criterion (AIC). The top 5 models with the lowest AIC were validated with the observed data points. 

### 3.3. Model Validation

The selected model was validated by the test dataset using the day forward-chaining cross validation method. The fitted model values were validated using the model performance analysis methods, including the Ljung–Box test, root mean squared error (RMSE), and Akaike information criterion (AIC). The Ljung–Box test is commonly used in ARIMA modeling to test whether the residuals of the fitted model have no autocorrelation. The Ljung–Box test uses the following hypotheses: H_0_: the residuals are independently distributed. H_A_: The residual exhibits serial correlation; they are not independently distributed. The test statistic (*Q)* can be defined as:

(5) $$Q=n\left(n+2\right)\;\overset{h}{\underset{k=1}{\sum }}\frac{{\hat{\rho }}_{k}^{2}}{n-k}$$

where *n* is the total number of data points, ${\hat{\rho }}_{k}^{}$ is the autocorrelation of lag *k*, and *h* is the number of lags being tested. The test statistic *Q* follows a chi-square distribution with *h* degree of freedom; that is, $Q\;\sim \;X^{2}\left(h\right)$. The null hypothesis is rejected when the residual of the fitted model values is not independently distributed; that is, $Q>X_{1-\alpha ,h}^{2}$ where $X_{1-\alpha ,h}^{2}$ is the (1−α)-quantile of the chi-squared distribution with the *h* degree of freedom at a significant level (α = 0.05).

The mean average percentage error (MAPE) was used to assess the regression error from the prediction value, as described in the equation below:

(6) $$MAPE=\frac{100\%}{n}\;\sum \left|\frac{y-\hat{y}}{y}\right|\;$$

where *n* is the total number of data points, *y* is the observed value, and $\hat{y}$ is the forecasted value from the fitted model. In this study, we used MAPE to assess the magnitude or significance of errors for the fitted model values. The accuracy evaluation of the model using the MAPE value can be interpreted using the table below (Table 1) [28]:

The model with a non-statistically significant *p*-value (*p* > 0.05) of the Ljung–Box test (Q18) with the lowest RMSE and MAPE was chosen to make 28-day forecasting for COVID-19 daily confirmed cases, recovery cases, and deaths. To analyze the performance and the accuracy of the final model, the 7-day forward chaining cross validation was considered. The test set was split temporally into a block of 7 days. The model validation method was repeatedly validated block by block. The final forecasted value was generated as well as the model performance measure parameters, including RMSE, MAPE, and U1 statistics. Theil’s U1 statistics is a commonly used statistic to measure the accuracy of the model; U ranges from 0 to 1 (0 ≤ U ≤ 1), when U closer to 0 indicates a higher forecasting accuracy [29]. Theil’s U1 statistics can be performed using the equation:

(7) $$U=\sqrt{\frac{1}{n}\overset{n}{\underset{t=1}{\sum }}e_{t}^{2}}/\left(\sqrt{\frac{1}{n}\overset{n}{\underset{t=1}{\sum }}y_{t}^{2}}+\sqrt{\frac{1}{n}\overset{n}{\underset{t=1}{\sum }}{\hat{y}}_{t}^{2}}\right)$$

where *y* is the observed value, $\hat{y}$ is the forecasted value, and *e* = $y-\hat{y}$ denotes a forecasted error. The further 28-day cumulative cases, case fatality rate, and mortality rate are generated and visualized by the results of the best-fitted final model. 

## 4. Results

**Figure 3:**
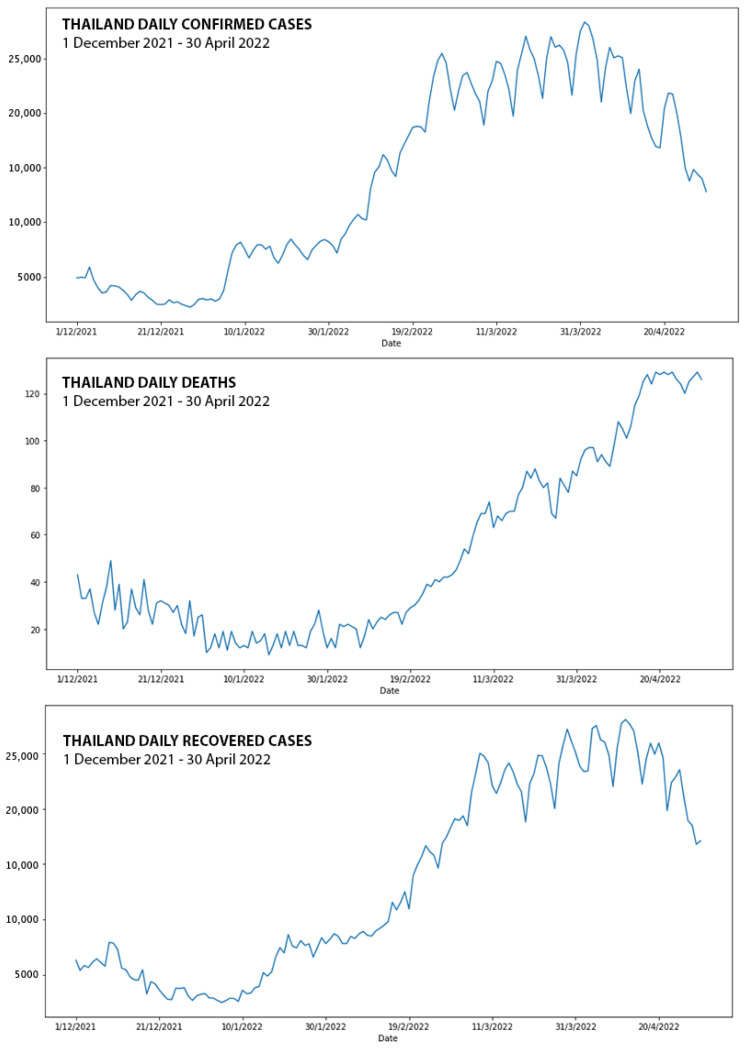
Graphical presentation of COVID-19 confirmed cases in Thailand during Omicron variant outbreak from 1 December 2021 to 30 April 2022.

The visualization of the overall time-series dataset that was used in this study showed that the COVID-19 confirmed cases in Thailand rapidly increased from the beginning of December 2021, as shown in Figure 3. Starting from mid-December 2021, COVID-19 patients were constantly confirmed with the Omicron variant, which dominated the old variants. The number of daily confirmed cases progressively rose for at least four continuous months, starting from December 2021. The augmented Dickey–Fuller (ADF) method was performed with the original datasets, and the first order of differencing for the stationarity validation is shown in Table 2. The unit root test for each dataset shows non-stationary characteristics for every dataset, except for the daily deaths with the first degree of differencing. 

### 4.1. Model Development

**Figure 4:**
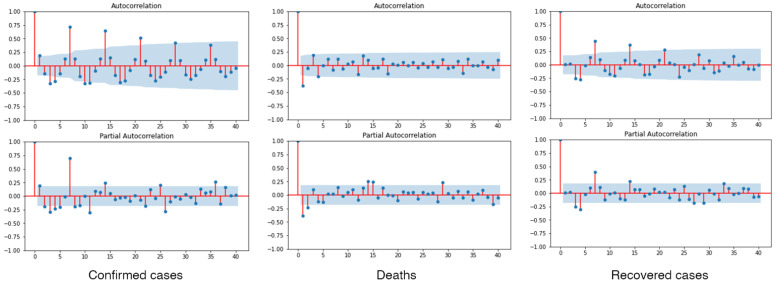
The ACF and PACF diagnostic plots after performing the 1st differencing order of the dataset with 95%CI.

In the process of selecting the proper forecasting models, the hyperparameters were estimated using the parameters grid search method based on the lowest AIC. The PACF and ACF diagnostic plots were examined to detect the trend and seasonality of the dataset. Some significant spikes at lags 7 and 14 were observed, which indicates that the dataset presented a seasonality every 7 days, as seen in Figure 4. This finding indicates that the seasonal trend can be observed for the Omicron outbreak in the Thailand dataset.

In this study, seasonal ARIMA (SARIMA) models were developed. The hyperparameters grid searching was performed based on the ACF and PACF plots. Overall, 36 combinations of SARIMA(p,d,q),(P,D,Q,s) models were developed within the following range: p, P = range (0,3); q, Q = range (1,3); d, D = range (1,2); and s = 7. The 36 combinations of SARIMA(p,d,q),(P,D,Q,s) models were performed with the training dataset and ranked by AIC results. The best five models out of 36 combinations from each dataset that gave minimum AIC were selected for a validation process.

### 4.2. Model Identification and Validation 

To identify the best performance model for validation using the validation dataset, the models with a non-statistically significant *p*-value of the Ljung–Box test, the lowest AIC, and the lowest RMSE were selected to generate a 28-day prediction. For the COVID-19 daily confirmed cases dataset, the SARIMA(1, 1, 1),(0, 1, 1, 7) was selected as a best fitted model for the validation process with the lowest AIC = 1336.32; RMSE = 1931.46; MAPE = 7.26%; Ljung–Box Q = 0.14 (*p*-value 0.71); and no heteroskedasticity. For the COVID-19 daily death dataset, the SARIMA(0, 1, 2),(1, 1, 1, 7) was selected as a best fitted model for the validation process with AIC = 754.13; RMSE = 8.90; MAPE = 5.82%; Ljung–Box Q = 0.05 (*p*-value 0.82); and no heteroskedasticity. For the COVID-19 daily recovered dataset, the SARIMA(0, 1, 1),(1, 1, 1, 7) was selected as a best fitted model for the validation process with the AIC = 1410.43; RMSE = 3686.54; MAPE = 12.13%; Ljung–Box Q = 0.09 (*p*-value 0.76); and no heteroskedasticity. These models were chosen as the best-performing model for the final validation. The validation results of the model diagnostic test for the COVID-19 daily confirmed cases, deaths, and recovered cases are shown in Table 3. 

**Figure 5:**
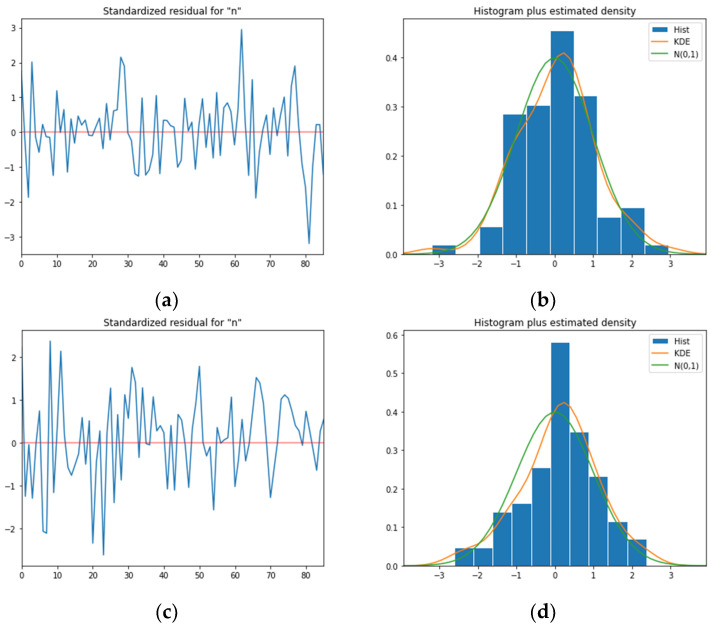

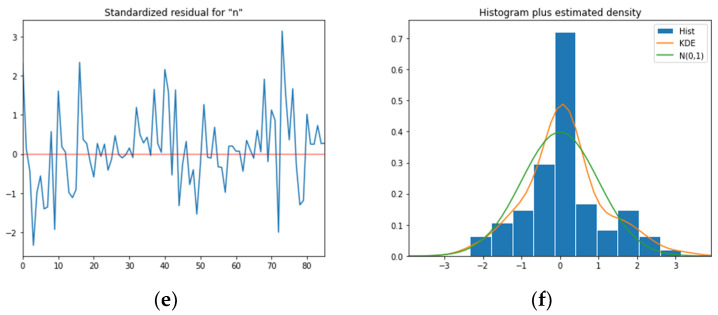
The standardized residuals plot (**left**), and a histogram and estimated density plot (**right**) for the selected models of daily COVID-19 confirmed cases (**a**,**b**); death (**c**,**d**); and recovered cases (**e**,**f**); within 1 standard deviation from the mean prediction value.

The diagnostic plots that are obtained from the model validation process are shown in Figure 5. The residual plots present a uniform variance pattern, as the errors are normally distributed with a mean zero. The estimated density of the mean prediction value suggests normal distribution, which indicates that the selected model is not overfitting, and the model is considered valid.

### 4.3. Model Performance Evaluation Results

**Figure 6:**
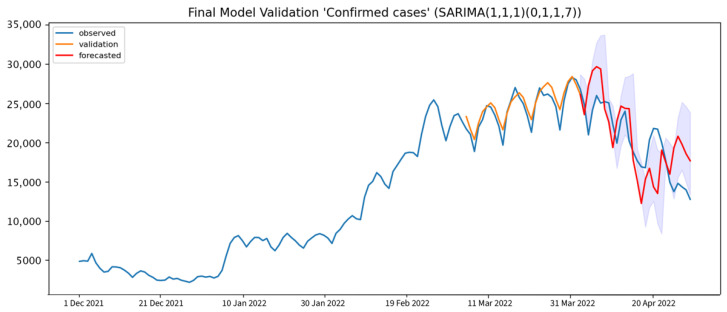
Model validation results of daily COVID-19 confirmed cases (orange solid line), compared to the observed cases and 7-day interval forecast daily COVID-19 confirmed cases (red solid line) with 95% CI (blue shade).

The forecasted values that were generated from the final models were assessed to compare the model performance and accuracy, as shown in Table 4. The model performance evaluation result shows that the SARIMA(1, 1, 1),(0, 1, 1, 7) model for COVID-19 confirmed cases forecasting has a 17.69% average MAPE when performing a forecasting every 7 days, which is considered good forecasting, compared to the 14-day interval and 28-day interval forecasting. The average Theil’s U1 statistic is 0.10, 0.13, and 0.22, respectively for 7-day, 14-day, and 28-day interval forecasting. The rapid downtrend during the last 7 days of the prediction might have affected the model. Figure 6 shows that the forecasted value is generated from the final model at a 7-day interval, which is the best time interval to forecast the COVID-19 daily confirmed cases in Thailand. Even though the validation plot visualization shows a good fit between the forecast and the observed data points, from the forecasted value, we can observe a seasonal lag of approximately 5–7 days. However, the observed lag does not affect the overall prediction performance.

The average RMSE and MAPE for the 7-day forward-chaining validation are 3799.57 and 17.69%, respectively. An average MAPE of less than 20 indicates good forecasting generated by the model. The average RMSE and MAPE for the 14-day forward-chaining validation are 5282.51 and 24.30%, respectively. The U1 statistic is 0.13. The average RMSE and MAPE for the 28-day forward-chaining validation are 7808.69 and 63.51%, respectively. The U1 statistic is 0.29.

**Figure 7:**
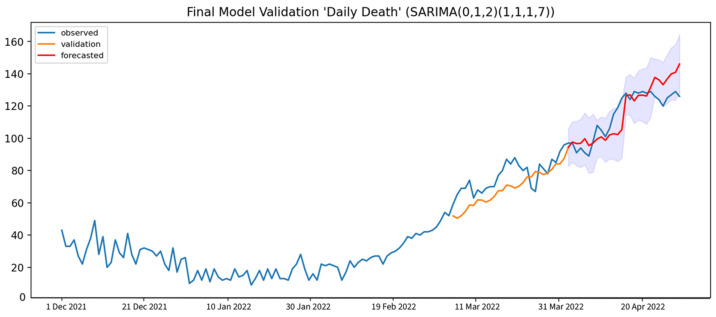
Model validation results of daily COVID-19-related deaths (orange solid line), compared to the observed cases and 7-day interval forecast daily COVID-19-related deaths (red solid line) with 95% CI (blue shade).

The forecasting of daily COVID-19 deaths was generated from the SARIMA(0, 1, 2),(1, 1, 1, 7) model at the 7-day interval with the average RMSE, MAPE, and U1 statistics of 8.25, 7.41%, and 0.04, respectively. Both the MAPE and U1 statistics for this forecasting indicated high accuracy and a good performance model as shown in Table 5. The prediction result that was generated from the selected model was plotted against the observed data point, as shown in Figure 7. The forecasted value is well aligned with the observed values. For the COVID-19 daily death dataset, a constantly rising trend can be observed. This makes the forecasting precise and accurate, even with a 28-day interval forecasting period, whose RMSE, MAPE, and Thiel’s U1 statistics are equal to 8.90, 5.82%, and 0.04 respectively.

The forecasting of daily COVID-19 recovered cases was generated from the 7-day interval SARIMA(0, 1, 1),(1, 1, 1, 7), which is the best performance model for capturing the COVID-19 daily recovered cases. The results are shown in Table 6. The 7-day interval forecasting results show the RMSE, MAPE, and U1 statistic values of 2484.41, 10.26%, and 0.0302, respectively. The 14-day interval forecasting has a 15.40% MAPE with Thiel’s U1 statistic at 0.08, which indicates good forecasting. The MAPE of the 28-day interval forecasting is 28.41%, which is reasonable, but the large-scale errors in this interval would make this 28-day interval ineffective for the prediction of COVID-19 daily recovered cases.

**Figure 8:**
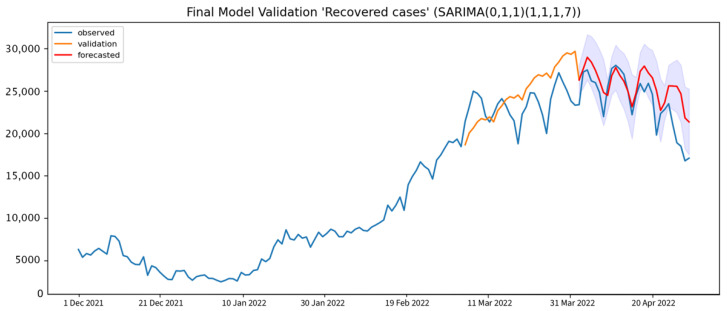
Model validation results of daily COVID-19 daily recovered cases (orange solid line), compared to the observed cases and 7-day interval forecast daily COVID-19 recovered cases (red solid line) with 95% CI (blue shade).

The prediction result that is generated from the selected model does not seem to capture the seasonal trend of the observed data during the validation phase. However, the final forecasting result shows that the SARIMA model needs to learn at least 4–5 seasonal cycles to capture the seasonality and trend of the time series. The forecasted value is a little higher than the observed value but still falls within the 95% CI, as shown in Figure 8.

### 4.4. Forecasting Trend of Thailand COVID-19 Daily Statistics

**Figure 9:**
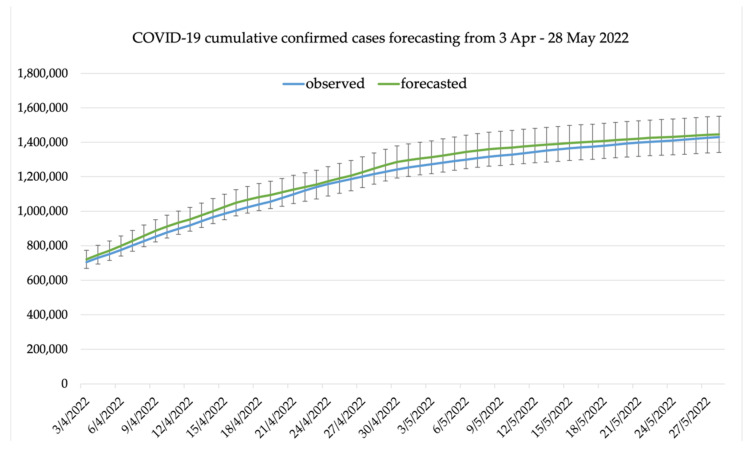
A forecasting result of cumulative COVID-19 cases in Thailand compared to the observed cumulative confirmed cases with mean average percentage error.

**Figure 10:**
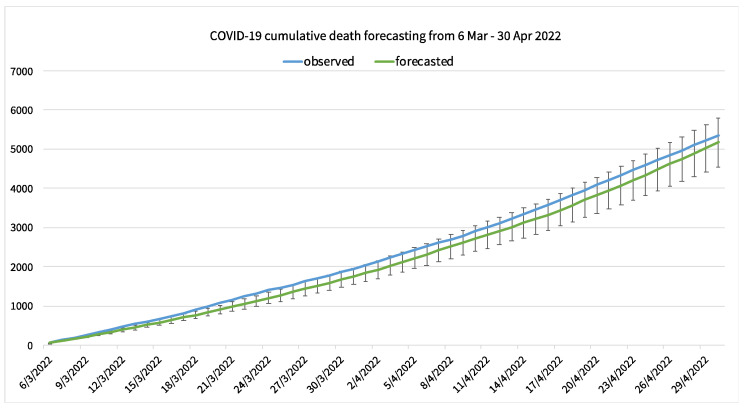
A forecasting result of COVID-19 cumulative deaths in Thailand compared to the observed cumulative deaths with mean average percentage error.

**Figure 11:**
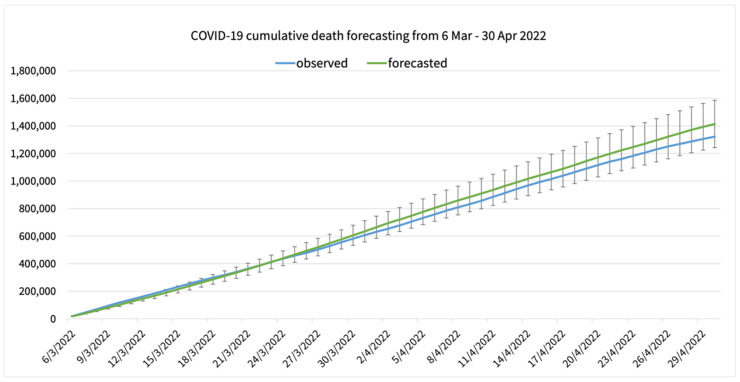
A forecasting result of COVID-19 cumulative recovered cases in Thailand compared to the observed cumulative deaths with mean average percentage error.

The cumulative daily COVID-19 confirmed cases, deaths, and recovered cases were calculated from the 7-day interval forecasted value and compared with the observed values. Figure 9 shows the observed cumulative COVID-19 confirmed cases in Thailand compared with the forecasted cumulative COVID-19 confirmed cases. The first 14-day forecasted cumulative confirmed cases is better compared to the latter 14-day forecasting. The 28-day cumulative death is shown in Figure 10, and the 28-day cumulative recovered cases is shown in Figure 11.

In this study, the case fatality rate and mortality rate of the COVID-19 Omicron variant epidemic were estimated for Thailand at the national level. These epidemiological parameters help us to determine the severity of the outbreak. The case fatality rate represents the ratio of patients with a COVID-19 confirmed diagnosis to patients who die because of the disease. Mortality rate can be interpreted as the risk of dying for patients that are diagnosed with COVID-19 infection. The case fatality rate and mortality rate per 100,000 population were calculated from the forecasted COVID-19 daily confirmed cases and deaths values.

**Figure 12:**
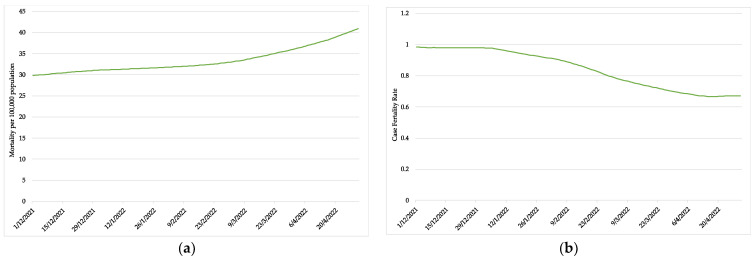
The forecasted COVID-19 mortality analysis of Thailand; (**a**) COVID-19 mortality rate per 100,000 population; (**b**) COVID-19 case fatality rate.

The trend of the case fatality rate and mortality rate that was forecasted from the aforementioned SARIMA models is shown in Figure 12.

## 5. Discussion

The time-series forecasting of the COVID-19 cases using the ARIMA model is currently the most straightforward method to help manage outbreak control in the future, given only one parameter time series. Even though there are published studies using ARIMA to predict the COVID-19 cases series, they scarcely describe the hyperparameter estimation process clearly, and the model validation remains questionable. The proper method of hyperparameter estimation is crucial when applying the ARIMA or SARIMA model to any of the time-series data, as well as the simplicity of the model when it is used in the forecasting process. The SARIMA model takes the seasonality as a parameter in a model because it is more powerful than ARIMA in forecasting the daily statistics of the COVID-19 Omicron variant epidemic, in which the seasonality pattern is clearly observed within the dataset.

To the best of our knowledge, this is the first study to develop SARIMA models to predict COVID-19 cases during the Omicron variant outbreak. The forecasted values were used to calculate the case fatality ratio and mortality rate. The model selection was based on the AIC value, which particularly compared how well the model fit the given data, while taking the complexity of the model into account. The models with the lowest AIC were selected because they showed a better fit, while keeping fewer features that made the model simpler. The top five models with the lowest AIC were validated with the observed data points. However, we found that the model with the lowest AIC result did not always give the smallest RMSE and MAPE. Thus, among the top five models, the one with the lowest RMSE and MAPE was chosen to make a 7-day, 14-day, and 28-day forward-chaining validation and forecast.

In this study, we described multiple time-series models that presented high forecasting accuracy to predict the daily COVID-19 confirmed cases, deaths, and recovered cases in Thailand using the government open dataset from 1 December 2021 to 30 April 2022, which covered the COVID-19 outbreak of the Omicron variant. A total of 151 data points were split into two contiguous parts for model training (1 December 2021 to 2 April 2022) and 28 data points were used as a validation dataset (3 to 30 April 2022). The best-fitted model for daily COVID-19 confirmed cases is a 7-day interval forecasting from SARIMA (1, 1, 1),(0, 1, 1, 7). The mean average error of predicted values (MAPE) is 17.69%, which is considered good forecasting for the time-series prediction, indicating that the model accuracy is good for COVID-19 daily confirmed cases forecasting. The daily COVID-19 confirmed cases data are a very important epidemiological factor that represent the dynamic and the severity of the outbreaks. In this study, the 7-day seasonal pattern of the daily COVID-19 confirmed cases is clear and strong. The 7-day peak pattern can be correlated with the average incubation period of the Omicron variant, which is quite stable in both seasonality and trend. The spike in the case number in each wave occurred either on a Tuesday or a Friday. In this case, we know that the incubation period of the COVID-19 Omicron variant ranges between 5 and 10 days, which is shorter than the Beta variant which led to the spike in numbers in the middle of the week. This phenomenon can be described as the higher infection rate that could happen over the weekend, while confirmed cases manifest later, after the incubation period. However, the rising trend of daily confirmed cases decreases and begins to enter the plateau phase, which indicates that the peak of the Omicron variant in Thailand is almost over and soon to begin a downtrend.

The forecasting of daily COVID-19 deaths and recovered cases were both generated from 7-day interval forecasting of SARIMA(0, 1, 2),(1, 1, 1, 7), and SARIMA(0, 1, 1),(1, 1, 1, 7), respectively. The calculated MAPE for both models was 6.35% and 10.27%, which was considerably low for the SARIMA prediction. The daily COVID-19-related deaths during the Omicron variant epidemic were the deaths among unvaccinated high-risk patients, pediatrics, and the elderly. The fluctuation in the daily number of deaths and recovered cases can be confounded by various exogenous factors that need further investigation.

Other important epidemiological parameters than the daily confirmed cases, deaths, and recovered cases are the case fatality rate and the mortality rate. The case fatality rate is the number of deaths among COVID-19 infected patients, while the mortality rate is the number of deaths among the total population within the area of interest. To calculate these parameters, the daily confirmed cases and confirmed deaths were used. From this study, we found that the case fatality rate decreased from 1% to nearly 0.6% during the Omicron variant epidemic, with a dramatic downward trend. The decreasing rate of case fatality indicates that the severity of the COVID-19 Omicron variant is lower than the previous variants.

However, the mortality rate per 100,000 population accumulatively increased during the Omicron variant epidemic. When the rate of infection is high, especially for the COVID-19 Omicron variant, the infected cases and deaths correlate increasingly. This parameter is very important to determine if the outbreak is near its end. If the mortality rate decreases, it can indicate that the epidemic is coming towards its end.

Using the SARIMA model to predict the COVID-19 case dynamic has proven to be effective and accurate, as reported in this study. The SARIMA model seems to be a robust model for public health applications, but the key to the performance accuracy relies on the hyperparameters of the model and the time interval of the prediction. This study shows that the 7-day interval forecasting expresses the highest accuracy compared to the 14-day and 28-day intervals. To make an appropriate forecasting interval, seasonality and trend need to be extensively observed. For the time series with a stable trend and season, the longer time interval forecasting can be accurate. For the fluctuating time series, the shorter time interval might be more effective to make a forecast.

Further exploration of the SARIMA model hyperparameters estimation process should be conducted to extend the time interval forecast and improve the forecasting performance of emerging disease outbreaks using both the ARIMA and SARIMA models. Some studies reported the implementation of a stochastic SEIR (susceptible, exposed, infected, recovery) model to obtain the hyperparameters for the ARIMA model, in order to predict the cumulative COVID-19 cases, which provided a positive result [30]. The SIR-based Riccati module is another interesting approach for the application of COVID-19 prediction. The approach can determine the correlation of socio-cultural context that could affect the rate of infection [31].

Seasonal ARIMA as a predictive model is a powerful tool for public health informatics. In particular, forecasting the trend of disease outbreaks with seasonality is the major challenge within the area of public health. The COVID-19 Omicron variant epidemic is one of the applications that prove the SARIMA is effective to use as a prediction model. To make the most of predictive modeling in public health, human factors, and human behaviors-related models should be further explored to improve the performance of time-series forecasting that will make an impact on the public health decision support system.

## 6. Conclusions

This work presented the most accurate SARIMA model to forecast at least 28 days ahead of the current outbreak in Thailand, especially for the daily COVID-19 confirmed cases. The study pointed out the importance of different time interval forecasting that affected the model performance. This can help to provide public health policymakers with a certain period to prepare the resources and plan for the outbreak management within the 7–28-day timeframe. This study also shows that the proposed SARIMA model is a robust model to predict uncertain time-series datasets accurately without using additional complicated parameters. This model development method might be applied to future emerging infectious diseases, not only with COVID-19. Further study on new data that are dimensionally reduced for univariate statistical modeling or multivariate statistical modeling, as well as stochastic modeling, should be considered to improve the predictive modeling in COVID-19 statistical forecasting, with hopes to extend the time interval forecasting.

## Figures and Tables

**Table 1:** Model accuracy evaluation criteria using MAPE values.

MAPE (%)	Interpretation
<10	Highly accurate forecasting
10–20	Good forecasting
20–50	Reasonable forecasting
>50	Inaccurate forecasting

**Table 2:** Unit root test results for training set using augmented Dickey–Fuller (ADF) method.

Dataset	Diff ^a^	ADF Value	*p* Value ^b^
Daily confirmed cases	0	0.31	0.98
	1	−1.94	0.31
Daily deaths	0	0.79	0.99
	1	−6.99	<0.01
Daily recovery cases	0	0.55	0.99
	1	−2.24	0.19

^a^ Number of differencing; ^b^ *p*-value less than 0.05 indicates that the dataset is stationary.

**Table 3:** The model training diagnostic results on the COVID-19 data performed by candidate models.

Model	AIC	RMSE	MAPE	Ljung–Box Test	
				Score	*p*-Value
**Confirmed cases**					
SARIMA(1, 1, 1),(0, 1, 1, 7)	1336.32	1931.46	7.26%	0.14	0.71
SARIMA(1, 1, 1),(0, 1, 2, 7)	1336.41	2230.31	8.18%	0.18	0.68
SARIMA(1, 1, 1),(1, 1, 1, 7)	1336.42	2281.49	8.31%	0.15	0.7
SARIMA(0, 1, 1),(1, 1, 1, 7)	1338.13	3185.02	11.28%	0.82	0.37
SARIMA(1, 1, 2),(0, 1, 1, 7)	1338.29	1999.01	7.44%	0.14	0.71
**Deaths**					
SARIMA(0, 1, 2),(1, 1, 2, 7)	756.04	8.93	5.87%	0.07	0.8
SARIMA(0, 1, 2),(1, 1, 1, 7)	754.13	8.90	5.82%	0.05	0.82
SARIMA(1, 1, 1),(1, 1, 2, 7)	756.57	9.27	6.22%	0.18	0.68
SARIMA(1, 1, 1),(1, 1, 1, 7)	754.30	8.99	5.87%	0.1	0.76
SARIMA(1, 1, 2),(1, 1, 2, 7)	758.35	9.01	5.91%	0.07	0.79
**Recovered cases**					
SARIMA(0, 1, 1),(0, 1, 1, 7)	1410.43	3686.54	13.51%	0.14	0.71
SARIMA(0, 1, 2),(0, 1, 1, 7)	1411.39	3799.29	13.90%	0.49	0.48
SARIMA(1, 1, 1),(0, 1, 1, 7)	1411.87	3750.77	13.74%	0.39	0.53
SARIMA(0, 1, 1),(1, 1, 1, 7)	1412.22	3312.99	12.13%	0.09	0.76
SARIMA(0, 1, 1),(0, 1, 2, 7)	1412.23	3343.85	12.25%	0.10	0.75

**Table 4:** The final model performance evaluation on the COVID-19 daily confirmed cases.

n-Day Forward Chaining	Block	Training Period	Forecasted Period	RMSE	MAE	MAPE (%)	U1
7-day interval	1	1 Dec 21–2 Apr 22	3–9 Apr 22	3097.87	2789.22	11.39	0.06
	2	1 Dec 21–9 Apr 22	10–16 Apr 22	2649.41	1733.54	8.66	0.06
	3	1 Dec 21–16 Apr 22	17–23 Apr 22	4936.84	4325.87	21.69	0.14
	4	1 Dec 21–23 Apr 22	24–30 Apr 22	4514.16	4099.44	29.02	0.13
			Average	3799.57	3237.02	17.69	0.10
14-day interval	1	1 Dec 21–2 Apr 22	3–16 Apr 22	5785.04	4890.97	22.30	0.11
	2	1 Dec 21–16 Apr 22	17–30 Apr 22	4779.98	4407.78	26.30	0.15
			Average	5282.51	4649.38	24.30	0.13
28-day interval	1	1 Dec 21–2 Apr 22	3–30 Apr 22	10,997.77	9429.39	55.08	0.22

**Table 5:** The final model performance evaluation on the COVID-19 daily deaths.

n-Day Forward Chaining	Block	Training Period	Forecasted Period	RMSE	MAE	MAPE (%)	U1
7-day interval	1	1 Dec 21–2 Apr 22	3–9 Apr 22	**4.88**	**4.01**	**4.37**	**0.03**
	2	1 Dec 21–9 Apr 22	10–16 Apr 22	11.45	9.59	8.27	0.05
	3	1 Dec 21–16 Apr 22	17–23 Apr 22	2.98	2.63	2.05	0.01
	4	1 Dec 21–23 Apr 22	24–30 Apr 22	13.68	13.40	10.70	0.05
			**Average**	**8.25**	**7.41**	**6.35**	**0.04**
14-day interval	1	1 Dec 21–2 Apr 22	3–16 Apr 22	7.19	5.35	5.02	0.03
	2	1 Dec 21–16 Apr 22	17–30 Apr 22	10.31	8.30	6.60	0.04
			**Average**	**8.75**	**6.83**	**5.81**	**0.04**
28-day interval	1	1 Dec 21–2 Apr 22	3–30 Apr 22	**8.90**	**6.90**	**5.82**	**0.04**

**Table 6:** The final model validation results on the COVID-19 daily recovered cases.

n-Day Forward Chaining	Block	Training Period	Forecasted Period	RMSE	MAE	MAPE (%)	U1
7-day interval	1	1 Dec 21–2 Apr 22	3–9 Apr 22	1999.88	1814.99	7.41	0.03
	2	1 Dec 21–9 Apr 22	10–16 Apr 22	748.85	663.08	2.57	0.01
	3	1 Dec 21–16 Apr 22	17–23 Apr 22	2523.33	1942.32	8.55	0.05
	4	1 Dec 21–23 Apr 22	24–30 Apr 22	4665.59	4235.41	22.55	0.10
			**Average**	**2484.41**	**2163.95**	**10.27**	**0.05**
14-day interval	1	1 Dec 21–2 Apr 22	3–16 Apr 22	2484.92	2296.17	9.15	0.04
	2	1 Dec 21–16 Apr 22	17–30 Apr 22	5238.33	4264.90	21.64	0.11
			**Average**	**3861.63**	**3280.54**	**15.40**	**0.08**
28-day interval	1	1 Dec 21–2 Apr 22	3–30 Apr 22	**7554.66**	**6023.39**	**28.41**	**0.14**

## Data Availability

The dataset used in this study was retrieved from Open Government Data of Thailand and Thailand National Center of Disease Control which are open for public analysis and can be downloaded from: https://data.go.th/en/dataset/covid-19-daily (accessed on 28 May 2022).
